# Carbamazepine treatment of bipolar disorder: a retrospective evaluation of naturalistic long-term outcomes

**DOI:** 10.1186/1471-244X-12-47

**Published:** 2012-05-23

**Authors:** Chia-Hui Chen, Shih-Ku Lin

**Affiliations:** 1 Department of Psychiatry, Taipei Medical University-Shuang-Ho Hospital, New Taipei City, Taiwan; 2 Department of Psychiatry, Taipei Medical University, Taipei, Taiwan; 3 Department of Psychiatry, Taipei City Hospital and Psychiatric Center, Taipei, Taiwan

**Keywords:** Bipolar disorder, Carbamazepine, Maintenance therapy

## Abstract

**Background:**

Carbamazepine (CBZ) has been used in the treatment of bipolar disorder, both in acute mania and maintenance therapy, since the early 1970s. Here, we report a follow-up study of CBZ-treated bipolar patients in the Taipei City Psychiatric Centre.

**Methods:**

Bipolar patients diagnosed according to the DSM-IV system and treated with CBZ at the Taipei City Psychiatric Centre had their charts reviewed to evaluate the efficacy and side effects of this medication during an average follow-up period of 10 years.

**Results:**

A total of 129 bipolar patients (45 males, mean age: 45.7 ± 10.9 year) were included in the analysis of CBZ efficacy used alone (n = 63) or as an add-on after lithium (n = 50) or valproic acid (n = 11), or the both of them (n = 5). The mean age of disease onset was 24.6 ± 9.5 years. The mean duration of CBZ use was 10.4 ± 5.2 year. The mean dose used was 571.3 ± 212.6 mg/day with a mean plasma level of 7.8 ± 5.9 μg/mL. Mean body weight increased from 62.0 ± 13.4 kg to 66.7 ± 13.1 kg during treatment. The frequencies of admission per year before and after CBZ treatment were 0.33 ± 0.46 and 0.14 ± 0.30, respectively. The most common side effects targeted the central nervous system (24%), including dizziness, ataxia and cognitive impairment. Other common side effects were gastrointestinal disturbances (3.6%), tremor (3.6%), skin rash (2.9%), and blurred vision (2.9%). Eighty-eight patients (68.2%) were taking antipsychotics concomitantly. Ninety-six patients (74.4%) needed to use benzodiazepines concomitantly. Sixty-three (48.8%) patients had zero episodes in a 10-year follow-up period, compared to all patients having episodes prior to treatment. Using variable analysis, we found better response to CBZ in males than in females.

**Conclusions:**

CBZ is efficacious in the maintenance treatment of bipolar disorder in naturalistic clinical practice, either as monotherapy or in combination with other medications. CBZ is well tolerated by most patients in this patient group.

## Background

Relapse in patients with bipolar disorder is a common problem. More than 90% of patients will have a recurrent episode, and it is also the cause of long-term morbidity and mortality [1,2]. Due to the chronic nature of this illness, there is a need for long-term maintenance treatment to prevent or attenuate the risk of relapse [3]. Lithium is the classic mood stabilizer used in treating acute and chronic bipolar disorder. However, studies have suggested that an estimated 20–40% of patients may have an inadequate prophylactic response to this drug [4,5]. Undesirable side effects and a narrow therapeutic index also make it less than ideal for many patients. This has led to efforts to investigate the potential utility of other mood stabilizers in maintenance therapy.

Carbamazepine (CBZ), the first anticonvulsant used in bipolar disorder, was recognized as a useful medication in the 1970s [6-8]. Moreover, it was found to be compatible with lithium in the treatment of acute mania in small-scale trials [9,10]. In Taiwan, CBZ has been increasingly used to treat bipolar disorder [11]. Its efficacy for treating acute mania was recently reconfirmed following the completion of large-scale, randomized, placebo-controlled trials in North America using extended-release CBZ [12,13].

Despite the widespread clinical use of mood-stabilizer combinations for the long-term treatment of patients with bipolar disorder, research on the risks and benefits of this practice is limited. In recent decades, only a few studies have focused on the use of CBZ for the maintenance of bipolar disorder [14-18]. The results of a 2-year follow-up study showed approximately equal effects when comparing CBZ and lithium in the prevention of mood episodes [18]. In a small-scale clinical trial comparing CBZ and placebo [19], the prophylactic effects of CBZ were superior in bipolar patients. However, several meta-analyses were unable to reach a conclusion about the efficacy of CBZ in prophylaxis [20-22]. In addition, there are a number of limitations when interpreting previous double-blind, controlled studies [23]. Firstly, most of these studies were based on a small sample size (up to 60 patients). Second, follow-up periods were relatively short (up to 2.5 years). Studies concerned with long-term efficacy tend to be more rigorous when their follow-up periods are longer. Unfortunately, studies that address the above-mentioned limitations are scarce. Therefore, we aimed to evaluate the clinical usage and long-term efficacy of CBZ in patients with bipolar I disorder treated with CBZ over an extended period. We reviewed the charts of a sample of 129 patients maintained on long-term CBZ therapy for up to 10 years. This paper describes the demographics, clinical characteristics and outcome data, and analyses the correlates of the outcome.

## Methods

### Setting and subjects

This was a retrospective chart-review study. Subjects comprised of patients attending the outpatient clinic of the Taipei City Psychiatric Center (TCPC) in Taipei, Taiwan. TCPC is a psychiatric hospital of 600 beds, and is one of the oldest and largest psychiatric hospitals in the Taipei metropolitan area. Most patients attending this hospital were referred from other general hospitals or clinics, and had been regularly followed up on. We included patients with a DSM-IV diagnosis of bipolar I disorder who had been treated at this hospital for at least 1 year with prophylactic CBZ (Tegretal®) either as monotherapy or combined with other mood stabilizers or antipsychotics. Diagnoses were made according to the medical records of the patient, and were usually made by at least two well-trained psychiatrists based on clinical evaluation and observation.

### Assessment

The study was based on a mirror-image study design, in which the clinical course under CBZ treatment was compared with data prior to CBZ treatment. The frequency of manic or depressive episodes and the frequency of hospitalization were recorded and analyzed as the main outcomes. We also collected clinically relevant data from the medical records and phone interviews, including demographic data, duration of illness and CBZ use, dosage and plasma concentration, concomitant medications, and reported side effects. The data were obtained by experienced research assistants, and the accuracy of recording was verified by two senior psychiatrists. Patients usually visited the clinic once a month but the frequency was dependent upon their clinical condition. An episode was defined as the recurrence of a full-blown manic or depressive mood event recorded in the chart. Patients who were prescribed with CBZ after their first episode were coded as ‘one episode prior to CBZ treatment’. The serum level of CBZ and other parameters were regularly monitored about twice a year, approximately 12 hours after the last dose of CBZ. Concomitant usage of medications other than CBZ was determined by clinical condition of the patient and was not limited due to the naturalistic nature of this study. We recorded the medications used at the last visit. We divided the patients into those with complete remission (CR) and those with non-CR, in which they had relapsed episode after CBZ treatment. This study was approved by the institutional review board of the hospital.

### Data analysis

The clinical variables were analyzed as mean ± SD. Paired *t*-test was utilized to analyze the changes in affective episodes before and after CBZ use. Independent *t*-test and Chi-square test were used to clarify correlations between variables and the treatment response.

## Results

### Characteristics of patients

A total of 129 (45 males) patients were recruited in this study. Table 1 shows the basic demographic and clinical characteristics of these patients. Their ages ranged from 22 to 80 years, but most (84.5%) were between 30 to 60 years old. Forty-four percent were married. Forty-one percent of the patients were in full-time or part-time employment, 23% were housewives, and 26% were unemployed. Most patients were judged to be regular attendees at follow-up and showed good drug compliance. The mean duration of disease course before CBZ treatment was 10.8 ± 7.6 years. Forty-two had started receiving CBZ after their first mood episode and 26 were switched to CBZ after receiving lithium or valproic acid. For the remainder, CBZ was used as an add-on to lithium (n = 45), valproic acid (n = 11), or the both of them (n = 5). Eighty-eight patients (68.2%) were taking low-dose antipsychotics concomitantly. Ninety-six patients (74.4%) needed to use benzodiazepines (mostly for the hypnotic action) concomitantly. CBZ monotherapy (without other mood stabilizer or antipsychotic) was used in 20 patients (15.5%). Among them, 12 patients were in the CR group. The patients’ mean body weight increased from 61.8 ± 13.2 to 66.6 ± 12.8 kg during treatment. The body weight increment was significantly less in patients treated with CBZ than those with combination of lithium, or valproic acid (3.41 ± 8.81 vs. 7.23 ± 10.60 kg, mean difference = −3.83, 95% CI = −7.64 to −0.01, p = 0.050), regardless of any combined use of antipsychotics. The mean number of admissions before and after CBZ was 2.26 ± 2.25 and 1.19 ± 1.80, respectively. After CBZ treatment, almost half of the patients (n = 63, 48.8%) had no further mood episodes during the follow-up period. Others (n = 66, 51.2%) suffered from varying frequencies of relapses ranging from 1 to 15 relapses.

**Table 1:** Clinical characteristics of study subjects (n = 129)

**Clinical characteristicsM**	**Mean ± SD**
Age (years)	45.7 ± 10.9
Age of onset (years)	24.6 ± 9.5
Education (years)	11.8 ± 3.3
Latency to CBZ treatment (Years)	10.8 ± 7.6
Follow-up period after CBZ treatment (Years)	10.4 ± 5.2
Current CBZ dose (mg)	571.3 ± 212.6
Current CBZ plasma level (μg/mL)	7.8 ± 5.9
Mood episodes before CBZ treatment	N (%)
1	42 (32.6)
2	22 (17.1)
3	28 (21.7)
4	16 (12.4)
≧5	21 (16.3)
Mood episodes after CBZ treatment	N (%)
0	63 (48.8)
1	21 (16.3)
2	22 (17.1)
3	6 (4.7)
≧4	17 (13.2)

*CBZ* carbamazepine.

### Effectiveness of CBZ

Table 2 shows the mean frequency (times per year) of hospitalization and mood episode pre- and post-CBZ treatment. Compared to the pre-CBZ treatment period, the post-CBZ period showed significantly fewer hospital admissions per year (p < 0.001), and fewer mood episodes per year (p = 0.002).

**Table 2:** Frequency (Mean ± SD times/year) and mean difference of hospitalization and mood episode before and after carbamazepine (CBZ) treatment

	**Before CBZ**	**After CBZ**	**Mean difference (95%CI)**	**P value**
Hospitalization frequency	0.33 ± 0.46	0.14 ± 0.30	0.18 (0.09, 0.27)	<0.001
Episode frequency	0.63 ± 1.70	0.16 ± 0.30	0.47 (0.17, 0.77)	0.002

*SD* standard deviation, *CI* confidence interval.

### Variables correlated with treatment response

We also delineated the correlates and characteristics of a good or poor response. Using our definition of CR and non-CR according to whether they had relapsed after CBZ treatment, 63 patients were placed in the CR group and 66 patients in the non-CR group. These two groups were then compared with respect to demographics and clinically-related variables. Data on the variables are presented in Table 3. The CR patients had a shorter follow-up duration after CBZ treatment (p = 0.046), a trend of fewer mood episodes before CBZ treatment (p = 0.054), and a higher likelihood of being male (p = 0.026).

**Table 3:** Comparison of clinical variables between subjects with/without complete remission (CR) by mean difference and relative risk (RR)

Variables	CR (n = 63)	without CR (n = 66)		
	Mean ± SD	Mean ± SD	Mean difference (95% CI)	P value
Latency to CBZ treatment (yrs)	11.86 ± 8.07	9.70 ± 6.96	2.16 (−0.47, 4.78)	0.106
Period after CBZ treatment (yrs)	9.49 ± 5.04	11.31 ± 5.23	−1.82 (−3.61, −0.03)	0.046
Episode before treatment	2.57 ± 2.13	3.32 ± 2.23	−0.75 (−1.51, 0.01)	0.054
Episode after treatment	0	2.65 ± 1.86	−2.65 (−3.11, −2.19)	<0.001
Drug dose (mg)	539.68 ± 187.98	601.52 ± 231.05	−61.83 (−135.42, 11.75)	0.099
Drug level (μg/mL)	7.22 ± 1.94	8.38 ± 8.03	−1.15 (−3.26, 0.95)	0.280
Age (yrs)	46.99 ± 11.17	44.55 ± 10.58	2.44 (−1.35, 6.23)	0.205
	N (%)	N (%)	RR (95% CI)	P value
Gender (female)	35 (55.6)	49 (74.2)	0.75 (0.58, 0.97)	0.026
Tobacco use	9 (14.5)	15 (22.7)	1.11 (0.94, 1.31)	0.234
Alcohol use n	5 (8.1)	5 (7.6)	1.00 (0.90, 1.10)	0918
Combined use of antipsychotics	36 (57.1)	52 (78.8)	2.02 (1.17, 3.49)	0.008
Combined use of BZD n	45 (71.4)	51 (77.3)	1.26 (0.70, 2.27)	0.447
Combined use of lithium	24 (38.1)	26 (39.4)	1.02 (0.78, 1.34)	0.880
Combined use of valproic acid	10 (15.9)	6 (9.1)	0.93 (0.81, 1.06)	0.243
Combined use of antidepressant	6 (9.5)	8 (12.1)	1.03 (0.91, 1.16)	0.635

*CBZ* carbamazepine, *SD* standard deviation, *CI* confidence interval, *BZD* benzodiazepine.

The most common side effects recorded on the chart were related to the central nervous system (24%), such as dizziness, ataxia, and cognitive impairment. Other common side effects were gastrointestinal disturbance (3.6%), tremor (3.6%), skin rash (2.9%), and blurred vision (2.9%).

## Discussion

To our knowledge, this is the longest outcome study that addresses the long-term use of CBZ in bipolar patients. We collected data from 129 patients exhibiting good compliance and attendance, which implies a reasonable quality of data. We used chart review to minimize the effect of recall bias, and all patients were evaluated by experienced clinicians. It is also worth noting that the long follow-up period (mean duration of 10 years) in this study could provide information that other studies lack. Mean plasma level of patients was 7.8 ± 5.9 μg/mL, which was within suggested therapeutic range for maintenance therapy.

We found that the frequency of mood episodes and hospitalization decreased significantly after CBZ treatment. We were also surprised to find that almost half of the patients (48.8%) had no more mood episodes after CBZ treatment, showing the efficacy of CBZ, either as monotherapy or combined with other mood stabilizers or antipsychotics, in the prophylactic treatment of bipolar disorder. It is difficult to compare our findings with other studies since there has been no other 10-year naturalistic follow-up of bipolar disorder patients treated with CBZ. The response rates for CBZ in previous studies varied. Kleindienst and Greil [24] found that classical bipolar patients treated with CBZ had a hospitalization rate of about 62% versus 26% in those treated with lithium in a randomized clinical trial with an observation period of 2.5 years. The better remission rate in our study might be related to the naturalistic design of the study, which allowed for a combination of other medications during the study period.

A second aim of our study was to determine the correlates of the CBZ prophylactic response. In previous studies, CBZ response was related to “non-classical disease”, and patients with suicidal behavior, lithium-refractory disease, and mixed episodes. However, in our study, no specific factors were correlated with the response to CBZ treatment with the exception that males had a better response rate. Since it was impossible to attain some of the clinical variables in this retrospective review, the response may be correlated with other factors that were not considered in this study.

The adverse events reported by the patients were also reviewed. The most common side effects coded were dizziness, fatigue and somnolence (24%). Most patients in our study were able to tolerate the side effects of long-term use of CBZ. Patients who did not receive concomitant lithium or valproic acid had significantly less body weight increment. This result confirms previous reports [14,21] suggesting that one benefit of CBZ include the low propensity toward weight gain and evidence of good tolerability with long-term treatment.

It is worth noting that about 40% of patients were treated concomitantly with lithium or valproic acid. Most patients needed benzodiazepines (74.4%) and antipsychotics (68.2%) in maintenance therapy. Given the naturalistic study design, we were unable to control the adjuvant medication. However, adjuvant medication usage did not differ between our CR and non-CR groups. In a review article by Keck and McElroy [25], the authors suggested that combination therapy may provide better long-term prevention of illness relapse and recurrence in many patients with bipolar disorder.

The main limitation of this study was the potential for a confounding bias due to its observational design. Since this study was designed as a retrospective chart review, it did not include patients who were initially treated with CBZ but discontinued use later for any reason. It was difficult to estimate the percentage of patients who dropped out of initial CBZ treatment, so the results should be interpreted with caution, and may only reflect the prophylactic effect in those who initially responded to acute treatment with CBZ. It is likely that patients who did not respond well to CBZ or who did not tolerate CBZ were more likely to drop out. In a 6-month, multicenter, open-label evaluation of beaded, extended-release CBZ capsule monotherapy in bipolar disorder patients with manic or mixed episodes [26], 68.8% of patients discontinued treatment early due to the lack of efficacy or side effects. This result reflects the fact that only a portion of patients adhere to CBZ according to perceived efficacy or tolerability of side effects. Our study merely showed that if a patient had a fair response to CBZ, they were likely to respond well in the continuing follow-up period. As mentioned earlier, due to the naturalistic setting, medications were not controlled during relapse. In addition, some variables were not reviewed (e.g., rapid cycling, or type of mood episode), which limited further analysis of clinical outcomes. Patients who were prescribed with CBZ during their first episode were coded as ‘zero episode prior to CBZ treatment’, which included 42 of the subjects. It is possible that patients with bipolar disorder who had just one episode over a long period of time may have a milder bipolar illness. However, after excluding these 42 subjects from analysis, the results remained the same (data not shown).

## Conclusions

As demonstrated, our longitudinal study proved that CBZ is efficacious and tolerable in the maintenance therapy of bipolar disorder in naturalistic clinical practice, either as monotherapy or in combination with other medications. Patients who have a good response to CBZ tend to continue to do well over the long term and their subsequent course of illness appears to be improved compared to their course of illness prior to CBZ treatment. This should be validated by more controlled long-term follow-up studies.

## Competing interests

The authors declare that they have no financial or other conflicts of interests in relation to this manuscript.

## Authors’ contributions

CHC and SKL designed the study, collected the data, and co-wrote the paper. Both authors read and approved the final manuscript.

## Pre-publication history

The pre-publication history for this paper can be accessed here:

http://www.biomedcentral.com/1471-244X/12/47/prepub
